# The kynurenine pathway activities in a sub-Saharan HIV/AIDS population

**DOI:** 10.1186/s12879-015-1087-5

**Published:** 2015-08-19

**Authors:** Priyesh Bipath, Peter F. Levay, Margaretha Viljoen

**Affiliations:** Department of Physiology, School of Medicine, Faculty of Health Sciences, University of Pretoria, Pretoria, South Africa; Department of Internal Medicine (Kalafong Hospital), School of Medicine, Faculty of Health Sciences, University of Pretoria, Pretoria, South Africa; Department of Psychiatry, School of Medicine, Faculty of Health Sciences, University of Pretoria, Pretoria, South Africa

## Abstract

**Background:**

Tryptophan is an essential amino acid for the synthesis of proteins and important metabolites such as serotonin, melatonin, tryptamine and niacin. After protein synthesis, more than 90 % of tryptophan catabolism occurs along the kynurenine pathway. The inflammation-inducible enzyme indoleamine 2,3 dioxygenase (IDO) is responsible for the first rate-limiting step in the kynurenine pathway, i.e., oxidation of tryptophan to kynurenine. Excessive IDO activity in conditions such as HIV/AIDS may lead to tryptophan depletion and accumulation of metabolites downstream from kynurenine. Little is known about the kynurenine pathway of HIV/AIDS patients in sub-Saharan regions. This study, in a low income sub-Saharan HIV/AIDS population, examined the effects of activities in the kynurenine pathway on plasma levels of tryptophan, kynurenine and the neurotoxin quinolinic acid, and on *de novo* synthesis of nicotinamide.

**Methods:**

Plasma samples were obtained from a cohort of 105 HIV patients and 60 controls. Kynurenine pathway metabolites were analysed using gas chromatography – mass spectrometry. ELISA and flow cytometry were used to assess plasma inflammatory markers.

**Results:**

IDO activity, depletion of tryptophan, as well as accumulation of kynurenine and the neurotoxin quinolinic acid, were not only significantly greater in the patients than in the controls, but also markedly greater than in HIV/AIDS patients from developed countries. Tryptophan levels were 12.3 % higher, kynurenine levels 16.2 % lower, quinolinic acid levels 43.2 % lower and nicotinamide levels 27,2 % lower in patients on antiretroviral treatment than in antiretroviral-naïve patients. Patients’ kynurenine pathway metabolites correlated with the levels of inflammatory markers, including that of the major IDO-inducer, interferon-gamma. Indications are that the rate of *de novo* synthesis of nicotinamide in the kynurenine pathway correlates with increases in quinolinic acid levels up to a point where saturation of the enzyme quinolinate phosphoribosyl transferase occurs.

**Conclusions:**

Higher levels of inflammatory activity in this low income sub-Saharan HIV/AIDS population than in patients from developed countries lead to greater tryptophan depletion and greater accumulation of metabolites downstream from tryptophan with quinolinic acid levels often reaching levels associated with the development of HIV/AIDS-associated neurocognitive dysfunction. *De novo* synthesis of nicotinamide from quinolinic acid contributes to the maintenance of nicotinamide, and by implication NAD levels, in HIV/AIDS patients from low income populations. Antiretroviral treatment partially corrects disturbances in the kynurenine pathway.

## Background

The essential amino acid tryptophan is important for protein synthesis and serves as substrate for the synthesis of serotonin, melatonin and tryptamine. In addition, it also serves as substrate for the *de novo* synthesis of nicotinamide adenine dinucleotide (NAD) and niacin in the kynurenine pathway of tryptophan metabolism. After protein synthesis, more than 90 % of tryptophan catabolism occurs along the kynurenine pathway [1].

**Fig. 1:**
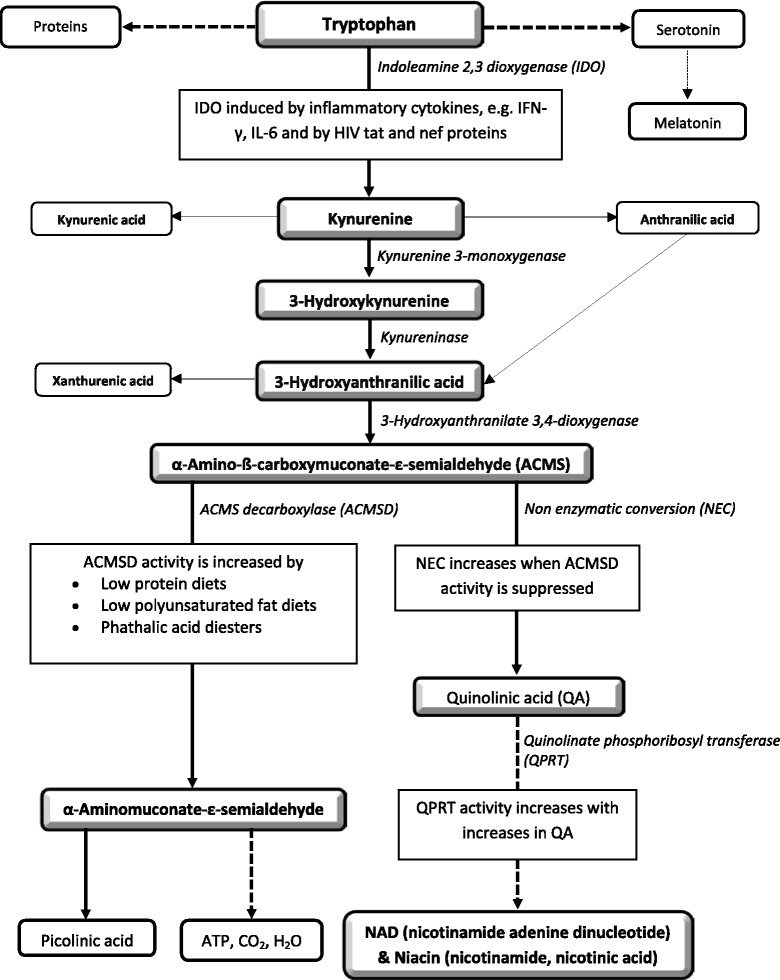
The kynurenine pathway

The kynurenine pathway starts with the oxidative degradation of tryptophan (Fig. 1). Kynurenine is the first stable metabolite formed when tryptophan is oxidized under influence of either L-tryptophan 2,3-dioxygenase (TDO) or indoleamine 2,3-dioxygenase (IDO) [2]. Excess tryptophan, i.e., at levels above the requirement for protein and serotonin synthesis, is oxidized in the liver under influence of the liver-specific enzyme TDO, to ATP, CO_2_ and water. In contrast, tryptophan oxidation under influence of the inflammation-inducible enzyme IDO occurs in various cell types, is not limited by a decrease in tryptophan levels and may even lead to tryptophan depletion [2]. The main cytokines for the induction of IDO are interferon-gamma (IFN-γ) in the periphery and interleukin-6 (IL-6) in the central nervous system, but other pro-inflammatory cytokines, as well as the HIV tat and nef proteins, may also have an influence [3, 4].

After conversion of tryptophan to kynurenine, kynurenine is converted to 3-hydroxy-kynurenine by the enzyme kynurenine 3-monoxygenase; 3-hydroxy-kynurenine is converted under the influence of kynureninase to 3-hydroxy-anthranilic acid and the latter converted under the influence of 3-hydroyxanthranilate 3,4-dioxygenase to α-amino-ß-carboxymuconate-ε-semialdehyde (ACMS) which, in turn, is converted to α-aminomuconate-ε-semialdehyde under the influence of the rate-limiting enzyme ACMS decarboxylase (ACMSD). Some ACMS, not metabolized to α-aminomuconate-ε-semialdehyde is non-enzymatically converted to quinolinic acid, the precursor of NAD and niacin (Fig. 1) [5–7].

Physical disorders such as autoimmune diseases, cancer, AIDS, pellagra, rheumatoid arthritis and cardiovascular abnormalities, as well as a host of neurodegenerative/neuropsychiatric disorders, have been linked to alterations in the kynurenine pathway of tryptophan metabolism [2, 4, 8]. Excessive activities in the kynurenine pathway, especially increases in quinolinic acid, are implicated in neurodegenerative disorders such as Alzheimer’s disease, Parkinson’s disease, Huntington’s disease, amyotrophic lateral sclerosis, schizophrenia and related disorders, multiple sclerosis, epilepsy, attention deficit-hyperactivity disorder, anxiety, depression and in the AIDS dementia complex [2, 8, 9]. Kynurenine pathway metabolites can either act as neuroactive substances or affect neuronal function through their oxidative/reductive properties, or through the supply of adequate NAD in conditions of a deficient dietary niacin [2, 8, 9]. Excessive stimulation of the kynurenine pathway may lead to tryptophan depletion, accumulation of the neurotoxin quinolinic acid and to a decline in serotonin synthesis [7–9].

Alterations in tryptophan metabolism along the kynurenine pathway in HIV/AIDS patients have previously been shown [10–17]. The majority of those studies were on populations from developed countries and primarily dealt with the first segment of the pathway, i.e., conversion of tryptophan to kynurenine. No previous study in HIV/AIDS patients could be found that simultaneously looked at the plasma levels of tryptophan, kynurenine, quinolinic acid, nicotinamide, as well as IDO activity, and the relevant immunological factors, and little is known about kynurenine pathway metabolism in HIV/AIDS populations from developing countries. The present study examined the effects of activities in the kynurenine pathway on tryptophan levels, on the accumulation of kynurenine and the neurotoxin quinolinic acid, and on the *de novo* synthesis of nicotinamide in a low income HIV/AIDS population from the Gauteng Province of South Africa. The findings are compared to that of HIV/AIDS patients from populations in developed countries.

## Methods

This cross-sectional study received approval, in accordance with the Declaration of Helsinki, from the Faculty of Health Sciences Research and Ethics Committee (Clearance Number 107/2008) of the University of Pretoria and from the hospital superintendent of Kalafong Hospital. Written or verbal informed consent was obtained from all participants. Patients unable to read or write were informed by a clinician about the nature and purpose of the study prior to obtaining verbal consent. The immunology clinic at the Kalafong secondary hospital in Pretoria was used as the research site for the recruitment of HIV positive patients. The immunology clinic provides health services to HIV positive patients from areas west of Pretoria, as well as from the surrounding townships. Patients are mostly of low socioeconomic status while many are unemployed or survive on a single grant or pension. Maize meal was reported as their staple food. An estimated 30 % of patients, attending the clinic, are foreigners from surrounding sub-Saharan African countries.

A total patient group of one hundred and five adult (>18 years of age) HIV positive patients (HIV-1, subgroup C) were voluntarily recruited at random during their scheduled visit to the clinic. HIV status was confirmed by the clinic which utilises testing performed by the National Health Laboratory Service (NHLS) at Kalafong. Of the total patient group 30 patients were not yet receiving highly active antiretroviral treatment (HAART), hereafter referred to as the HAART-naïve group, and 75 patients were already on HAART. Patient demographic information is given in Table 1. A total of 60 HIV negative controls (63 % female) with a mean age of 31.18 ± 8 .09 years and body mass index (BMI) of 21.96 ± 4.81 kg/m^2^ were also recruited from the South African National Blood Service (SANBS). Ethical clearance and approval were received from the SANBS Human Research Council (Clearance Number 2010/03) and written informed consent was obtained from all of the participants.

**Table 1:** Patient demographic information

	Total patients	HAART	HAART-naïve
n	105	75	30
Females	66 (63 %)	48 (64 %)	18 (60 %)
Age (years)	35.97 ± 9.58	37.86 ± 8.86	37.13 ± 10.24
Ethnicity	105 Black	75 Black	30 Black
Married	32 (30 %)	21 (28 %)	11 (37 %)
Employed	43 (44 %)	33 (44 %)	13 (43 %)
Smoker (≥1 cigarette per day)	20 (19 %)	13 (17 %)	7 (23 %)
Alcohol consumer (≥1 drink per week)	12 (11 %)	7 (9 %)	5 (17 %)
Body mass index - BMI (kg/m^2^)	23.16 ± 5.91	23.83 ± 6.31	20.96 ± 3.62
Average months on HAART	-	15.86 ± 16.49	-
Tuberculosis co-infection	24 (22.9 %)	14 (19 %)	10 (33 %)

Data expressed as mean ± SD

The plasma levels of tryptophan, kynurenine, quinolinic acid and nicotinamide were simultaneously determined by gas chromatography coupled to mass spectrometry (GC-MS) using the method which was developed and validated in our laboratory. Briefly, samples were processed and derivatized with pentaflouropropionic anhydride and pentaflouropropanol before analysis. The GC oven was programmed to begin at an initial temperature of 80 °C with a ramp at a rate of 20 °C up to 180 °C followed by a 10 °C ramp up to a maximum temperature of 280 °C. Sample peaks were eluted on a DB-5MS capillary column within a chromatographic runtime of 18 min using a Thermo Scientific Trace 1300 gas chromatographer coupled to an ISQ single quadropole mass spectrometer. Neopterin and cytokine levels were determined by enzyme linked immunosorbent assay (ELISA) and cytometric bead array flow cytometry respectively. All other variables were determined by the National Health Laboratory Service at Kalafong.

Data are expressed as mean and standard deviation. Groups were compared by ANOVA and subgroups by non-parametric Kruskal-Wallis. Associations between variables were tested by non-parametric Spearman rank correlation coefficients. All testing was performed at a significance level of *p* < 0.05 using SPSS (Version 22, IBM Inc.).

## Results

**Fig. 2:**
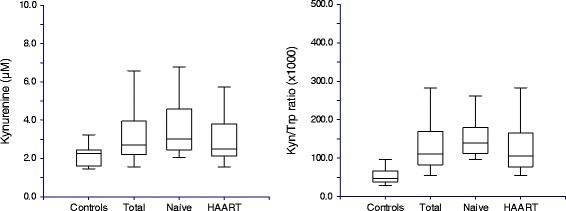
Box plots of kynurenine and K/T ratio for the control, total patient, HAART-naïve and HAART groups

The plasma levels for tryptophan, kynurenine, quinolinic acid, nicotinamide, neopterin, IFN-γ, IL-6, as well as the CD4 counts and the kynurenine/tryptophan ratios for the patient groups and the controls, are compared in Table 2. When patients and control groups were compared, tryptophan levels were 49.4 and 42.3 % lower, kynurenine levels 63.7 and 43.9 % higher, quinolinic acid levels 2208 and 1512 % higher, and nicotinamide levels 31.0 and 3.02 % higher for the HAART-naïve and HAART groups, respectively, than for the control group. Patients with CD4 counts below 200 cells/μl presented with significantly higher kynurenine levels (3.92 ± 1.54 vs. 2.87 ± 1.01; *p* = 0.002) and kynurenine/tryptophan (K/T) ratios (171.90 ± 78.11 vs. 116.41 ± 48.45; *p* = 0.001) than patients with CD4 counts greater than 200 cells/μl. Figure 2 shows the comparisons for kynurenine and the K/T ratios for the different groups. A search was performed to find all studies in which one or more metabolites of the kynurenine pathway have been measured in HIV positive patients. The results can be seen in Table 3.

**Table 2:** Comparison of the results between the patient groups and the control group

	Total Patients	HAART (H)	HAART-Naïve (N)	Controls (C)	*p*-value (H vs. N)	*p*-value (H vs. C)	*p*-value (N vs. C)
n	105	75	30	60	-	-	-
Tryptophan (μmol/l)	24.36 ± 4.14	25.13 ± 3.80	22.04 ± 4.32	43.57 ± 11.85	0.033	<0.001	<0.001
Kynurenine (μmol/l)	3.21 ± 1.33	3.08 ± 1.28	3.58 ± 1.42	2.14 ± 0.45	0.144	<0.005	<0.001
Quinolinate (μmol/l)	4.46 ± 2.32	4.03 ± 2.04	5.77 ± 2.65	0.25 ± 0.06	0.072	<0.001	<0.001
Nicotinamide (μmol/l)	14.25 ± 9.47	13.31 ± 9.65	16.93 ± 8.61	12.92 ± 3.69	0.108	0.773	0.046
K/T ratio (μM/mM)	136.03 ± 65.45	129.69 ± 65.36	158.07 ± 62.51	52.18 ± 16.95	0.095	<0.001	<0.001
Neopterin (nmol/l)	45.57 ± 41.82	35.51 ± 35.70	66.63 ± 40.73	8.23 ± 5.71	<0.001	<0.001	<0.001
IFN-γ (pg/ml)	44.00 ± 22.55	41.43 ± 14.14	53.68 ± 34.39	24.85 ± 2.96	0.017	<0.001	<0.001
IL-6 (pg/ml)	11.16 ± 14.95	9.56 ± 12.54	15.04 ± 19.34	0.69 ± 1.62	0.010	0.035	0.001
CD4 (cells/μl)	259.92 ± 195.32	296.21 ± 195.50	170.05 ± 167.26	-	0.003	-	-

Results expressed as mean ± SD

**Table 3:** Studies in which metabolites of the kynurenine pathway have been assessed in HIV patients

		Tryptophan (μmol/l)	Kynurenine (μmol/l)	K/T ratio	Quinolinic Acid (μmol/l)	^a^Niacin, nico- tinic acid or ^b^nicotinamide (μmol/l)	IFN-γ (pg/ml)
Sub-Saharan Countries							
Present Study, South Africa	Patients	24.36 ± 4.14	3.21 ± 1.33	136.03 ± 65.45	4.46 ± 2.32	^b^14.25 ± 9.47	44.46 ± 22.46
	Controls	43.57 ± 11.85	2.14 ± 0.45	52.18 ± 16.95	0.25 ± 0.058	^b^12.92 ± 3.69	24.85 ± 2.96
	p-value	<0.0001	0.0001	<0.001	<0.0001	0.198	<0.0001
Byakwaga et al. 2014 [20], Uganda	Patients	18	2.157	131	Not done	Not done	Not done
	Controls	Not done	Not done	Not done	Not done	Not done	Not done
Martinez et al. 2014 [21], Uganda	Patients	18.17	~2.22	122.2	Not done	Not done	Not done
	Controls	Not done	Not done	Not done	Not done	Not done	Not done
Developed Countries							
Fuchs et al. 1991 [11], Austria	Patients	57.0 ± 2.8	3.45 ± 0.14	-	Not done	Not done	259 ± 7 (U/l)
	Controls	91.0 ± 6.63	2.31 ± 0.23	-	Not done	Not done	23.5 (U/l)
	p-value	<0.01	<0.01	-	Not done	Not done	<0.01
Huengsberg et al. 1998 [12], Austria	Patients	50.1	2.55	50.5	Not done	Not done	Not done
	Controls	56.3	1.98	34.9	Not done	Not done	Not done
	p-value	<0.01	<0.001	<0.001	-	-	-
Look et al. 1998 [13], Germany	Patients	44.6	4.1	108.2	0.848	Not done	Not done
	Controls	52.6	2.7	51.4	0.303	Not done	Not done
	p-value	0.14	0.002	0.002	0.001	-	-
Zangerle et al. 2002 [14], Austria	Patients	44.1 ± 13.3	3.01 ± 0.91	79.2 ± 60.3	Not done	Not done	Not done
	Controls	65.8 ± 12.8	2.02 ± 0.66	30.7 ± 8.7	Not done	Not done	Not done
	p-value	<0.001	<0.001	<0.001	-	-	-
Schroeksnadel et al. 2008 [15], Austria	Patients	51.40	2.60	51.15	Not done	Not done	Not done
	Controls	Not done	Not done	Not done	Not done	Not done	Not done
Heyes et al. 1998 [29], USA	Patients	Not done	Not done	Not done	16.85 ± 3.36	Not done	Not done
Skurnick et al. 1996 [44], USA	Patients	Not done	Not done	Not done	Not done	^a^43.9 ± 0.89	Not done
	Controls	Not done	Not done	Not done	Not done	^a^37.4 ± 1.38	Not done
	p-value	-	-	-	-	0.0001	-
Heyes et al. 2001 [30], USA	Patients	Not done	Not done	Not done	1.358 ± 0.939	Not done	Not done
	Controls	Not done	Not done	Not done	0.416 ± 0.122	Not done	Not done
Bogden et al. 1990 [45], USA	Patients	Not done	Not done	Not done	Not done	^a^43.86 ± 2.44	Not done
	Controls	Not done	Not done	Not done	Not done	Not done	Not done

Results expressed as mean ± SD. *P*-values represent comparisons between patients and control values, where available. The superscripted symbol a denotes that niacin values were reported. The superscripted symbol b denotes that nicotinamide levels were reported.

**Fig. 3:**
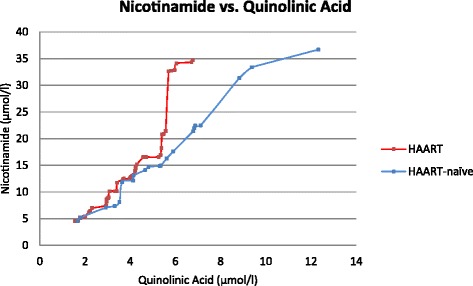
Relationship between nicotinamide and quinolinic acid levels. Information presented from ordered data sets (smallest to largest values)

Correlations were determined to delineate any positive or negative associations between the kynurenine pathway metabolites on the one hand and immune indicators on the other. The correlations for kynurenine levels, K/T ratio, quinolinic acid levels and nicotinamide levels with markers of immune activity are given in Table 4. Table 5 represents the comparison of neopterin levels between the present study and that of developed countries at corresponding levels of immune deficiency (CD4 counts). Figure 3 illustrates the relationship between nicotinamide and quinolinic acid for the HAART and HAART-naïve group.

**Table 4:** Correlations of kynurenine, K/T ratio, quinolinic acid and nicotinamide with CD4 counts, neopterin, IL-6 and IFN-γ

	Kynurenine (μmol/l)		K/T ratio		Quinolinic Acid (μmol/l)		Nicotinamide (μmol/l)	
Variable	Rho	p-value	Rho	p-value	Rho	p-value	Rho	*p*-value
CD4 count	−0.393	0.0008	−0.366	0.0027	−0.110	0.371	−0.082	0.516
Neopterin	0.514	<0.0001	0.538	<0.0001	0.309	0.0036	−0.014	0.904
IL-6	0.354	0.0007	0.362	0.0008	0.062	0.566	−0.112	0.317
IFN-γ	0.344	0.0010	0.366	0.0007	−0.030	0.781	−0.145	0.192

Spearman Rho correlations for the total patient group; significance at *p* < 0.05

**Table 5:** Neopterin levels in HIV patients from the present study and from populations in developed countries

	Total patient groups		At lower CD4 counts		At higher CD4 counts	
Study	NPT	CD4	NPT	CD4	NPT	CD4
Present Study	45.57	257.97 cells/μl	70.48	<200 cells/μl	24.07	>200 cells/μl
Zangerle et al. 2002 [14]	23.4	112 cells/μl	23.4	112 cells/μl	8.0	232 cells/μl
Schroeksnadel et al. 2008 [15],	14.05	404 cells/mm^3^	-	-	-	-
Mildvan et al. 2005 [53],	16.03	75 cells/ml	20.4	50 cells/ml	9.9	200 cells/ml
Hanna et al. 2009 [54],	-	-	24.4	<200 cells/μl	12.5	>200 cells/μl
Kurz et al. 2009 [55],	25.0	204 cells/mm^3^	-	-	-	-
Bogner et al. 1988 [56],	-	-	29.7	264 cells/μl	14.4	487 cells/μl

*NPT* Neopterin (nmol/l). CD4 count units given as cells per unit volume as indicated per publication

## Discussion

The study examined the effects of activities in the kynurenine pathway on the levels of tryptophan, kynurenine and quinolinic acid, as well as on *de novo* synthesis of nicotinamide in a low income HIV/AIDS population from the Gauteng Province of South Africa. The findings are compared to that of HIV/AIDS patients from populations in developed countries.

The total patient group was severely tryptophan depleted compared to the controls (24.36 ± 4.14 μmol/l vs. 43.57 ± 11.85 μmol/l; *p* < 0.0001) and the degree of tryptophan depletion correlated with the degree of immune deficiency (tryptophan vs. CD4: *r* = 0.341; *p* = 0.004). The tryptophan levels in patients on anti-retroviral treatment were significantly higher than those not yet on treatment (HAART 25.13 ± 3.80 μmol/l vs. HAART-naïve 22.04 ± 4.32 μmol/l; *p* = 0.03). Tryptophan levels were markedly lower in the population of this study than in HIV/AIDS patients from developed countries (Table 3).

The initial step was to examine the degree of oxidation of tryptophan to kynurenine in the first part of the kynurenine pathway as a possible contributor to the markedly lower tryptophan levels seen in this population than in HIV/AIDS patients from developed countries.

### Oxidation of tryptophan to kynurenine

In the first segment of the kynurenine pathway, tryptophan is converted to kynurenine under influence of the rate limiting enzyme IDO [2]. The results of this study showed that, despite significantly lower tryptophan levels in the patients than in the controls (24.36 ± 4.14 μmol/l vs. 43.57 ± 11.85 μmol/l; *p* < 0.001), kynurenine levels were significantly higher in the patients (3.21 ± 1.33 vs. 2.14 ± 0.45 μmol/l; *p* < 0.001). Although higher kynurenine levels were seen in the HAART-naïve group than the HAART group, the difference was not statistically significant (kynurenine: 3.58 ± 1.42 vs. 3.08 ± 1.28 μmol/l; *p* = 0.144). The finding of higher kynurenine despite lower tryptophan levels is in line with the fact that the activity of the IDO enzyme is, in contrast to TDO, not substrate dependent. The kynurenine/tryptophan (K/T) ratio is generally used as an indication of the activity of IDO [3, 4, 12, 15]. The K/T ratios were significantly higher (*p* < 0.001) in the patients than in the control group. The association between inflammatory activity and IDO activity was subsequently investigated. When the K/T ratios were compared to the levels of neopterin, a marker of inflammatory activity, and to that of the pro-inflammatory cytokine IL-6, significant positive correlations were found, both between the K/T ratios and neopterin (*r* = 0.514; *p* < 0.0001) and between the K/T ratio and IL-6 (*r* = 0.354; *p* = 0.00071). This is in agreement with the view of pro-inflammatory activity being the main stimulus for the conversion of tryptophan to kynurenine [3, 4, 7, 9], in other words the major stimulus for IDO activation. Perhaps of more importance is the significant positive correlation found between the K/T ratios and the levels of IFN-γ (*r* = 0.344; *p* = 0.001), the pro-inflammatory cytokine considered to be the primary IDO inducer [3, 4].

When the values of our patient group were compared to values obtained for HIV/AIDS patients from developed countries, the IDO activities, as indicated by the K/T ratios, were markedly higher in the patients from our population (Table 3). However, inflammatory activity was also much higher at comparable levels of immune deficiency (Table 5). These findings of higher K/T ratios and higher pro-inflammatory activity in our population compared to populations from developed countries, coupled to the highly significant positive associations found between the K/T ratios and inflammatory activity, especially IFN-γ (neopterin: *r* = 0.514; *p* < 0.0001; IL-6: *r* = 0.354; *p* = 0.0007; IFN-γ: *r* = 0.344; *p* = 0.001), and the negative associations between IFN-γ and tryptophan (*r* = −0.217; *p* = 0.036), strongly suggest higher levels of inflammatory activity, leading to a higher rate of tryptophan oxidation in the kynurenine pathway, to be a major contributor to the lower tryptophan levels found in the black, low income, sub-Saharan population of this study than in HIV/AIDS populations from developed countries. In view of the reported high incidence of clinical and/or subclinical infections and malnutrition in low income sub-Saharan populations [18] and the fact that malnutrition further stimulates inflammatory activity [19], findings of a higher inflammatory activity at comparable CD4 counts comes as no surprise.

The frequency of tuberculosis is one example of infections that may potentially contribute to higher rates of inflammatory activity in our HIV/AIDS patients, and thus to higher rates of tryptophan degradation. In this study 24 of the 105 patients were diagnosed with tuberculosis. However, they were all already on treatment (isoniazid, pyrazinamide, rifampicin, and ethambutol) for tuberculosis. Although no significant differences were found for either tryptophan (*p* = 0.591) or IFN-γ levels (*p* = 0.432) between the TB-positive and the TB-negative of the total group, a potential bias was present in the fact that 14 TB-positive patients were from the HAART group (19 % of 75), and 10 TB-positive patients were from the HAART-naïve group (33 % of 30). Further investigation is necessary to assess the influence of TB-co-infection, preferably including HIV/AIDS TB-positive patients not yet on treatment for tuberculosis.

Shortly after completion of this study, information emerged on the first segment of the kynurenine pathway (tryptophan conversion to kynurenine) in an Ugandan population (Table 3) [20, 21]. This appears to be the first data published on the kynurenine pathway in an African population. The results of the Ugandan project and that of the present study are in agreement with regards to the findings of higher kynurenine levels and K/T ratios in patients from resource-limited settings than in HIV populations from developed countries. Inflammatory activity was unfortunately not assessed in their study. As in the Ugandan study, associations were seen in the present study for CD4 counts with kynurenine levels and with the K/T ratios (Table 4). This could perhaps be seen, as was suggested by the authors of the Ugandan paper [20], as an association between kynurenine pathway activity and immune deficiency or disease progression and mortality. However, it is more likely a reflection of the association between the higher inflammatory activity with a decline in immune deficiency (neopterin vs. CD4 count: *r* = −0.558; *p* < 0.0001; Il-6 vs. CD4 count: *r* = −0.435; *p* = 0.00012; IFN-γ vs. CD4: *r* = −0.271; *p* = 0.02), coupled to the influence of inflammatory activity on IDO activity (Table 4). Such negative associations between inflammatory activity and CD4 counts and positive associations between inflammatory activity and disease progression have been demonstrated by us in a previous paper [22].

### Kynurenine to quinolinic acid

Downstream from kynurenine in the kynurenine pathway, quinolinic acid is synthesized from ACMS (Fig. 1). The largest part of ACMS is metabolized to α-aminomuconate-ε-semialdehyde under influence of the rate-limiting enzyme ACMSD. ACMS, not converted to α-aminomuconate-ε-semialdehyde, is non-enzymatically converted to quinolinic acid [5–7]. ACMSD thus determines the amount of ACMS available for conversion to quinolinic acid and decreased ACMSD activity could therefore increase ACMS turnover towards quinolinic acid [6, 23, 24]. The activity of ACMSD is known to be down-regulated by diets low in protein and low in poly-unsaturated fatty acids [24, 25]. ACMSD activity is also inhibited by dietary phthalic acid diesters, leading to an increase in quinolinic acid synthesis and in the conversion ratio of tryptophan to niacin [25, 26]. Various enteric-coated medications are said to contain phthalates, including the antiretroviral Didanosine [27].

Quinolinic acid is found in micromolar concentrations in the plasma and reported normal values are usually in the range of 0.2 to 0.5 μmol/l [13, 28, 29, 30]. However, it is known to increase with several immune-associated disorders and quinolinic acid levels several times normal have been reported in cerebrospinal fluid (CSF) and plasma in neurological conditions such as Alzheimer’s disease, Parkinson’s disease and other neurocognitive and psychiatric disorders [2, 30, 31, 32].

In the present study plasma quinolinic acid levels for the control group corresponded to published values for normal. However, quinolinic acid levels were significantly higher for the total patient group than for the controls (4.46 ± 2.32 vs. 0.25 ± 0.058 μmol/l; *p* < 0.0001). The difference in quinolinic acid levels between the HAART (4.03 ± 2.04 μmol/l) and the HAART-naïve groups (5.77 ± 2.65 μmol/l) bordered on significance (*p* = 0.072). In line with previous studies [13, 30], the present study thus showed lower quinolinic acid levels in patients on HAART than in HAART-naïve patients. When quinolinic acid levels were compared to markers of pro-inflammatory activity, significant positive correlations were seen with neopterin for the total patient group (*r* = 0.309; *p* = 0.0036) and for the HAART group (*r* = 0.249; *p* = 0.041). The HAART-induced decline in quinolinic acid production was most probably related to the partial correction of the pro-inflammatory/anti-inflammatory balance brought about by anti-retroviral medication (HAART-naïve neopterin vs. HAART neopterin: 66.63 ± 40.73 vs. 35.51 ± 35.70 nmol/l). As for kynurenine, quinolinic acid levels increased with increased immune deficiency. Patients with CD4 counts below 200 cells/μl presented with higher quinolinic acid levels as compared to patients with CD4 counts above 200 cells/μl (5.13 ± 2.67 μmol/l vs. 3.98 ± 2.02 μmol/l; *p* = 0.052). This increase in quinolinic acid levels with increased immune deficiency was most likely the result of the increased inflammatory activity that accompanied immune deficiency.

Comparing the quinolinic acid plasma values of our study to values obtained for HIV/AIDS patients elsewhere proved to be difficult. It would appear that only a few groups in developed countries studied plasma quinolinic acid as part of the kynurenine pathway in HIV patients, whereas no such study could be found on patients from sub-Saharan Africa (Table 3). In the present study quinolinic acid levels were 21.1 fold higher in the HAART-naïve patients and 16.1 fold higher in the HAART patients than in the controls. The increments above control values were much higher than that found by Heyes et al. 2001 [20], and by Look et al. 2000 [13], in populations from developed countries (Table 3).

Several factors probably contributed to the high levels of quinolinic acid synthesis seen in our HIV/AIDS population. Firstly, ACMS, the precursor of quinolinic acid, levels are dependent on the activities in the kynurenine pathway upstream from quinolinic acid, especially the levels of kynurenine produced by the oxidative catabolism of tryptophan [24]. The high levels of kynurenine found in this study were discussed in a previous paragraph. Secondly, in addition to the increase in the upstream substrate levels for ACMS synthesis, there might have been a shift in the metabolism of ACMS in favour of the non-enzymatic conversion to quinolinic acid. Such a shift, previously ascribed to an immune-induced suppression of ACMSD expression, has previously been reported in primary cultures of human macrophages stimulated by IFN-γ [33]. Thirdly, the fact that ACMSD activity can also be down-regulated by diets low in proteins and polyunsaturated fats [9, 24, 25] could have been a contributing factor in the population of the present study where, in the majority of patients, maize represented the staple food. Although the phthalate-containing enteric-coated antiretroviral medication Didanosine [27], which is on code at the hospital attended by the HIV/AIDS population of this study, can suppress ACMSD activity, it is not as a rule prescribed and can thus in most patients be ruled out as a contributor to the high quinolinic acid levels.

Support for the occurrence of quinolinic acid at levels as high as those found in the present study were derived from studies on neurocognitive function in HIV/AIDS patients. Quinolinic acid has previously been implicated in AIDS dementia and a number of studies measured quinolinic acid in cerebrospinal fluid of HIV/AIDS patients [34–35]. A few of these studies measured it in CSF, as well as in plasma [36, 37]. At least one such study [37] found plasma quinolinic acid levels (4.041 ± 0.892 μM) comparable to that of the present study (4.46 ± 2.32 μmol/l). From papers where quinolinic acid were measured in both plasma and CSF it would appear that a correlation exists between plasma and CSF levels, but that plasma quinolinic acid levels are up to 10 times higher than in CSF [36]. As shown by the present study for plasma (QA vs. neopterin: *r* = 0.309; *p* = 0.0036), quinolinic acid levels in CSF were reported to correlate with immune activity as indicated by neopterin levels [22, 36, 37].

Serious neurological/psychiatric effects such as in inflammatory brain disorders and in the AIDS dementia complex have been reported with CSF quinolinic acid levels in the range of 0.5 to 1.2 μmol/l [31, 32, 30]. In the present study plasma quinolinic acid levels ranged between 1.56 and 12.33 μmol/l. In view of the latter, the high quinolinic acid levels of this study, primarily resulting from excessive inflammatory activity, do not augur well for the neuropsychiatric wellness of HIV/AIDS patients from the population of the present study.

### Quinolinic acid to nicotinamide

The term niacin is the generic name for the two compounds, nicotinic acid and nicotinamide, the major precursors for NAD [38, 39]. Nicotinamide is said to be the predominant and biologically active form of niacin in circulation, with nicotinic acid, after absorption, being converted to nicotinamide by hepatocytes [38, 39]. Diet-derived nicotinamide, as well as nicotinic acid can be metabolised to NAD, although in slightly different pathways [9, 45]. Although niacin is generally described as a vitamin, niacin and NAD can also be synthesized in the kynurenine pathway. Tryptophan is the primary substrate for this *de novo* synthesis with quinolinic acid as the direct precursor of niacin and NAD (Fig. 1) [6, 39]. While it has in the past been assumed that only about 2 % of niacin is derived from *de novo* synthesis, there are indications that the contribution is of much greater importance [6]. Synthesis from tryptophan has even been suggested as the primary source of NAD and niacin [9].

Several papers refer to a niacin/NAD deficiency in HIV/AIDS patients [7, 38, 40–42]. However, no clinical study has actually proved niacin deficiency in HIV patients without pellagra. Instead of assessment of plasma niacin or nicotinamide levels, deficiency is generally assumed by the appearance of symptoms of pellagra and occasionally by the determination of metabolites of niacin excreted in the urine [41]. However, the incidence of pellagra in HIV/AIDS is probably overestimated. Pitche P et al., 1999, for instance, found the incidence of pellagra and pellagra-like erythema in HIV patients in Togo not to be higher than that in the general population [43]. Two groups seem to have actually measured the plasma or serum levels of niacin in HIV patients, one in 1990 and the other in 1996 [44, 45]. Both studies reported niacin levels to be higher in their HIV positive groups than in their controls.

The patient population of the present study were generally of low income or unemployed with some families surviving on a single grant or pension. Maize meal, known to be an inadequate source of niacin [46, 47] was the staple diet of the population. It would thus not be unreasonable to expect niacin levels to have been subnormal. A number of patients were, however, prescribed B complex supplementation comprising 40 mg nicotinamide per day (two B.CO tablets/day; 20 mg nicotinamide per tablet; European RDA ≤ 700 mg/day, based on side effects). The international guideline for daily niacin supplementation, established by the Food and Nutrition Board, ranges from 14 to 18 mg with an upper intake level of 35 mg [48].

In the present study nicotinamide levels were numerical higher for the total patient (HAART plus HAART-naïve) group than for the controls, but this difference was not statistically significant (14.25 ± 9.47 vs. 12.92 ± 3.69 μmol/l; *p* = 0.198). However, although the difference between the HAART and HAART-naïve patients were not of statistical significance (13.31 ± 9.65 vs. 16.93 ± 8.61 μmol/l; *p* = 0.108), nicotinamide levels were significantly higher in the HAART-naïve patients compared to the controls (16.93 ± 8.61 vs. 12.92 ± 3.69 μmol/l; *p* = 0.046). None of the patients showed signs of pellagra on clinical examination.

Despite the fact that niacin supplementation was only prescribed for some of the patients and despite strong reservations about patient compliance (personal communication), it must be assumed that supplementation contributed to the nicotinamide levels in at least some of the patients studied. The question does, however, arise whether *de novo* synthesis made a significant compensatory contribution for the dietary deficiencies, as well as the absorption problems and increased NAD utilisation, previously reported for HIV/AIDS patients [49, 50]. Plasma nicotinamide levels were therefore correlated to that of the precursor, quinolinic acid. In Fig. 3 the relationship between plasma nicotinamide and quinolinic acid is shown. Nicotinamide levels increased with increases in quinolinic acid concentration up to a plasma level of 5 μmol/l in HAART patients and up to about 8 μmol/l in HAART-naïve patients after which hardly any further increases occurred. Significant positive correlations were seen between nicotinamide and quinolinic acid concentrations for the HAART patients up to a plasma level of 5 μmol/l (*r* = 0.545; *p* = 0.0001) and for the HAART-naïve patients up to 8 μmol/l (*r* = 0.882; *p* < 0.0001). These significant positive correlations suggest a substantial portion of the circulating nicotinamide to be derived from *de novo* synthesis from quinolinic acid.

Quinolinate phosphoribosyl transferase (QPRT) is the rate limiting enzyme in the synthesis of NAD, nicotinamide and nicotinic acid from quinolinic acid. Although QPRT activity is known to increase in response to increases in the levels of quinolinic acid [51], it is also known that conversion of quinolinic acid to NAD and nicotinamide is, in the human central nervous system, limited by saturation of QPRT [52]. Neuronal QPRT activity is saturated when quinolinic acid concentration exceeds 500 nM (0.5umol/l), and it is suggested that this may play a role in the toxic accumulation of quinolinic acid [52]. It was therefore of interest to know whether QPRT saturation also limits the conversion outside the central nervous system. From the levelling-off in niacin levels seen in Fig. 3 it would appear that QPRT becomes saturated at plasma levels around 5 μmol/l in patients on HAART and just above 8 μmol/l in HAART-naïve patients. Of interest is the fact that, according to the literature, quinolinic acid are on average up to 10 times higher in plasma than in CSF [36], which would be in line with the observation that nicotinamide levels started to level off in plasma at quinolinic acid levels 10 times higher than that reported for CSF.

## Conclusions

This is the first study to assess plasma tryptophan levels, kynurenine levels, IDO activity, quinolinic acid levels and nicotinamide levels, as well as pro-inflammatory status and IFN-γ levels, simultaneously in one HIV/AIDS population. Patients of the present study were all from a black, low income sub-Saharan population where malnutrition and higher rates of clinical and subclinical infections are bound to have had an influence.

The results of this study showed that higher levels of inflammatory activity, at comparable levels of immune deficiency, contributed to a higher degree of tryptophan depletion in this low income sub-Saharan population than in populations from developed countries. This, as shown in the results, contributed to higher levels of kynurenine pathway metabolites such as kynurenine and quinolinic acid. Largely due to the high inflammatory activity, but ostensibly also due to the effects of dietary insufficiencies on ACMSD activity, quinolinic acid levels were above the saturation level for QPRT activity and, for several patients, within the range associated with the development of HIV/AIDS-associated neurocognitive dysfunction. Associations between quinolinic acid and nicotinamide levels suggested a sizeable contribution of the kynurenine pathway to the maintenance of nicotinamide, and by implication NAD, in patients with HIV/AIDS. Antiretroviral treatment partially corrected disturbances in the kynurenine pathway.

## Abbreviations

ACMS: α-amino-ß-carboxymuconate-ε-semialdehyde
ACMSD: α-amino-ß-carboxymuconate-ε-semialdehyde decarboxylase
BMI: Body mass index
CSF: Cerebrospinal fluid
ELISA: Enzyme linked immunosorbent assay
GC-MS: Gas chromatography mass spectrometry
HAART: Highly active antiretroviral treatment
IDO: Indoleamine 2,3 dioxygenase
IFN-γ: Interferon-gamma
IL: Interleukin
K/T: Kynurenine/tryptophan
NAD: Nicotinamide adenine dinucleotide
QPRT: Quinolinate phosphoribosyl transferase
TDO: Tryptophan 2,3-dioxygenase
